# 3D-embedding of human gingiva-derived mesenchymal stromal cells in “VitroGel^®^ MSC” hydrogel alters their immune mediator expression under inflammatory conditions

**DOI:** 10.1186/s12903-026-08473-6

**Published:** 2026-05-21

**Authors:** Katharina Schwarz, Oliwia Miłek, Merjem Bećirović, Christian Behm, Karl Heinrich Schneider, Oleh Andrukhov

**Affiliations:** 1 Competence Center for Periodontal Research, University Clinic of Dentistry, Medical University of Vienna, Vienna, Austria; 2 Center for Biomedical Research and Translational Surgery, Medical University of Vienna, Vienna, Austria

**Keywords:** Mesenchymal stromal cells, Gingiva, 3D culture, Hydrogel, Immunomodulation

## Abstract

**Background:**

Human gingiva-derived mesenchymal stromal cells (hG-MSCs) are promising candidates for cell-based therapy due to their immunomodulatory ability. Culturing in 3D hydrogels has emerged as a promising strategy to improve cell survival in the host and enhance clinical efficacy. This study assesses how embedding hG-MSCs in “VitroGel^®^ MSC” hydrogel affects their immunomodulatory profile in various inflammatory environments, compared with conventional 2D cultures.

**Methods:**

hG-MSCs from six donors were either 3D-embedded in commercially available “VitroGel^®^ MSC”, cultured on “VitroGel^®^ MSC”, or in 2D on tissue culture plastic under resting conditions or in the presence of interferon (IFN)-γ, interleukin (IL)-1β, or tumor necrosis factor (TNF)-α. Metabolic activity and cell viability were assessed by MTT assay and live/dead staining, respectively. Gene expression of IL-8, cyclooxygenase (COX)-2, indoleamine-2,3-dioxygenase (IDO)-1, programmed cell death ligand 1 (PD-L1), PD-L2, and TNF-stimulated gene (TSG)-6 were analyzed by RT-qPCR, and IL-8 and prostaglandin E_2_ (PGE_2_) protein levels were determined by ELISA.

**Results:**

Metabolic activity was reduced in 3D-embedded hG-MSCs independently of the environment, but only in the presence of IFN-γ and TNF-α, a slight decrease in cell viability was observed. Hydrogel-embedded and -exposed hG-MSCs exhibited increased IL-8 expression under resting and IFN-γ conditions, and consistently upregulated COX-2 and PGE_2_. The immunomodulatory mediator TSG-6 was elevated in 3D-embedded hG-MSCs, while the expression of IDO-1, PD-L1, and PD-L2 upon transition to 3D hydrogel was differently altered depending on the inflammatory environments.

**Conclusions:**

These results demonstrate that 3D microenvironments reshape hG-MSC immunomodulatory potential, underscoring the relevance of 3D models for optimizing MSC-based therapies.

**Supplementary Information:**

The online version contains supplementary material available at 10.1186/s12903-026-08473-6.

## Background

Over the last decades, mesenchymal stromal cells (MSCs) have attracted growing attention in regenerative medicine due to their therapeutic potential [1]. These cells possess the ability to self-renew [2] and to differentiate in vitro into multiple mesenchymal lineages, including adipocytes, chondrocytes, and osteoblasts [3]. Additionally, MSCs are known for their immunomodulatory functions [4] by expressing various immune mediators, including indoleamine 2,3-dioxygenase-1 (IDO-1), programmed death ligands 1 and 2 (PD-L1, PD-L2), prostaglandin E_2_ (PGE_2_), cyclooxygenase-2 (COX-2), and tumor necrosis factor-α stimulated gene 6 (TSG-6) [5]. These mediators are differentially regulated in response to pro-inflammatory cytokines [6], such as interferon-γ (IFN-γ), interleukin-1β (IL-1β), and tumor necrosis factor-α (TNF-α), allowing MSCs to rapidly respond to their environment and dynamically interact with the immune system [7].

MSCs can be isolated from various human sources, including dental tissues, such as the dental pulp [8], periodontal ligament [9], and gingiva [10]. In particular, human gingival tissue is easily accessible and often discarded as clinical waste [11], making human gingiva-derived mesenchymal stromal cells (hG-MSCs) a convenient and valuable resource. Moreover, hG-MSCs originate from the neural crest [12], showing stable morphology, sustained MSC marker expression with increased passages, and enhanced proliferation whilst maintaining a normal karyotype, telomerase activity [13], and being non-tumorigenic [14]. Their effectiveness has been highlighted in pre-clinical and clinical studies focusing on the treatment of oral and craniofacial disorders [11, 12], including gingival defects [14], maxillofacial bone defects [15, 16], periodontal disease [17, 18], and peri-implantitis [19, 20]. Specifically, hG-MSCs promote wound healing by facilitating re-epithelialization [21] and regulating the inflammatory response [22]. Additionally, the secretion of various pro- and anti-inflammatory mediators by hG-MSCs in response to inflammatory cytokines and bacterial components supports tissue homeostasis and is relevant for inflammatory conditions, such as periodontitis and peri-implantitis [23].

Despite positive outcomes reported in numerous pre-clinical studies, clinical studies have shown limited therapeutic success [1] due to various factors, including poor cell survival and retention after transplantation [24]. Preserving the secretory and immunomodulatory functions of MSCs after transplantation remains crucial for therapeutic efficacy. To overcome these challenges, various improved cell delivery systems have been developed so far [19, 20], with one promising approach being the embedding of MSCs in hydrogels. Hydrogels are versatile polymeric materials, derived from natural, synthetic, or hybrid sources, which can be chemically modified to meet specific needs [1]. One commercially available hydrogel is “VitroGel^®^ MSC”, a polysaccharide-based injectable hydrogel, which has already been used in in vivo studies [25, 26]. Embedding of MSCs in hydrogel provides a physiologically relevant 3D microenvironment that significantly influences cell behavior [27]. Compared to conventional 2D culture, 3D-embedding alters cell adhesion, spreading, migration, and polarity. Moreover, it generates a gradient of paracrine factors, and more closely mimics the lower mechanical stiffness of native tissues. Previous studies have shown that basal MSCs cultured in 3D exhibit increased paracrine activity [28], as indicated by the elevated expression of PGE_2_ and TSG-6 [29] by bone marrow-derived MSCs. Similarly, 3D-cultured adipose-derived MSCs exhibited increased expression of tryptophan 2,3-dioxygenase (TDO2) [30], an enzyme similar to IDO-1 that is responsible for tryptophan catabolism [31]. Despite growing interest in the immunomodulatory effects of MSCs in 3D cultures, no studies have specifically addressed this topic in gingiva-derived MSCs under pro-inflammatory conditions.

Hence, this study investigates how 3D-embedding using “VitroGel^®^ MSC” influences the expression of mediators involved in the inflammatory and immunomodulatory functions of hG-MSCs in pro-inflammatory environments. For this purpose, hG-MSCs 3D-embedded in the hydrogel were compared to those cultured on the hydrogel and on conventional tissue culture plastic (TCP), under basal conditions and in the presence of IFN-γ, IL-1β, or TNF-α. The results of this study provide new insights into how hydrogel-based 3D microenvironments affect the immunomodulatory abilities of hG-MSCs, which could contribute to the optimization of MSC-based therapies in regenerative medicine.

## Methods

### Experimental design

This study compared the influence of 3D and 2D culture conditions on key immune mediator expression of human gingiva-derived mesenchymal stromal cells (hG-MSCs) in pro-inflammatory conditions. For this purpose, three experimental groups were established: hG-MSCs embedded within hydrogel (3D in hydrogel), hG-MSCs cultured on top of the hydrogel to evaluate the effect of the hydrogel material itself (on hydrogel), and hG-MSCS cultured in conventional 2D conditions on tissue culture plastic (2D on TCP).

### Rheometric evaluation of “VitroGel^®^ MSC”

Rheometric properties of the hydrogel “VitroGel^®^ MSC” (TheWell Bioscience, North Brunswick Township, NJ, USA) were analyzed using a MCR 301 rheometer equipped with a 25 mm parallel-plate PP25/P3 measuring system and controlled via the RheoCompass™ software (all from Anton Paar, Graz, Austria). “VitroGel^®^ MSC” was mixed in a ratio of 1:3 with high glucose Dulbecco’s Modified Eagle’s Medium (DMEM; Sigma-Aldrich, St. Louis, MO, USA) supplemented with 30% fetal bovine serum (FBS; Capricorn Scientific, Ebsdorfergrund, Germany), 300 U/mL penicillin and 300 µg/mL streptomycin (Capricorn Scientific, Ebsdorfergrund, Germany) to obtain a final concentration of 10% FBS, 100 U/mL penicillin, and 100 µg/mL streptomycin. 1 mL of the prepared gel was immediately loaded onto the plate, and the zero gap was set to 2 mm. The dynamic time sweep was immediately performed at 24 °C, 1% shear-strain and 1 rad/s for 60 min. The cyclic compression test was performed 30 min. after gel polymerization by applying 10% compressive strain for 10 cycles. The stiffness of the hydrogel was calculated based on the averaged slopes of the last three compression cycles.

### hG-MSC isolation and cultivation

The isolation protocol was approved by the Ethics Committee of the Medical University of Vienna (No. 1079/2019, valid until 03/2027). All experiments were conducted in accordance with the Declaration of Helsinki and the Medical University of Vienna’s “Good Scientific Practice” guidelines. Primary hG-MSCs were isolated as previously described [32] from the gingiva attached to extracted molars from healthy donors (two male and four female), who had undergone extraction for orthodontic reasons and gave their written consent. In brief, the extracted tooth was washed with phosphate buffered saline (PBS, pH 7.2) (Capricorn Scientific, Ebsdorfergrund, Germany) and transferred to a 10 cm CytoOne petri dish (Starlab International, Hamburg, Germany) filled with DMEM (10% FBS, 100 U/mL penicillin, and 100 µg/mL streptomycin). The gingiva was dissected from the tooth, minced, and left to culture in the prepared petri dish at 37 °C, 5% C_2_, and 95% hmidity. The media was replaced once a week, until the outgrowing hG-MSCs reached 80% confluence. Thereafter, hG-MSCs were passaged using Accutase solution (Sigma-Aldrich, St. Louis, MO, USA) and expanded in 175 cm² cell culture flasks (Greiner Bio-One International, Kremsmünster, Austria) under the aforementioned culture conditions. hG-MSCs with passages 2–7 were used for all experiments. The correspondence of hG-MSCs to the minimal criteria of MSCs [33, 34] by the International Society for Cell and Gene Therapy was confirmed as previously described [35].

### hG-MSC hydrogel cultures

Seeding of hG-MSCs in “VitroGel^®^ MSC” was performed according to the manufacturer’s protocol. First, a cell suspension was prepared by resuspending hG-MSCs in DMEM (30% FBS, 300 U/mL penicillin and 300 µg/mL streptomycin). The prepared cell suspension was mixed 1:3 with “VitroGel^®^ MSC” to obtain a final concentration of 10% FBS, 100 U/mL penicillin, and 100 µg/mL streptomycin. 150 µL of this cell-hydrogel mixture was transferred to a 48-well plate (VWR, Radnor, PA, USA) per well with a final concentration of 7.5 × 10⁴ hG-MSCs per hydrogel. After 30 min of polymerization at room temperature, the hydrogel was covered with 150 µL of DMEM (10% FBS, 100 U/mL penicillin and 100 µg/mL streptomycin) and incubated for 24 h at 37 °C.

hG-MSCs on hydrogel and on TCP served as material- and 2D-controls, respectively. To seed the cells on the hydrogel, DMEM (30%, FBS, 300 U/mL penicillin and 300 µg/mL streptomycin) was mixed 1:3 with “VitroGel^®^ MSC” to obtain a final concentration of 10% FBS, 100 U/mL penicillin, and 100 µg/mL streptomycin. After 30 min of polymerization at room temperature, 2.3 × 10⁴ hG-MSCs were seeded on top of each hydrogel in 150 µL DMEM (10% FBS, 100 U/mL penicillin and 100 µg/mL streptomycin). To seed cells on TCP, 2.3 × 10⁴ hG-MSCs in 150 µL DMEM (10% FBS, 100 U/mL penicillin and 100 µg/mL streptomycin) were seeded directly into the wells. Both controls were incubated for 24 h at 37 °C.

24 h after seeding, hG-MSC cultures were washed with PBS and either left unstimulated (basal) or were stimulated with pro-inflammatory cytokines (100 ng/mL IFN-γ, 5 ng/mL IL-1β or 10 ng/mL TNF-α) (PeproTech, London, UK) [6] in 300 µL DMEM (2% FBS, 100 U/mL penicillin and 100 µg/mL streptomycin). After 24 h of incubation at 37 °C, supernatants were stored at -80 °C for ELISA (0–24 h), and hG-MSC cultures were stimulated again as described above for another 24 h at 37 °C.

### Metabolic activity analysis

Thiazolyl blue tetrazolium bromide (MTT; Sigma-Aldrich, St. Louis, MO, USA) was added directly to the culture media in the wells to achieve a final concentration of 0.5 mg/mL, and was incubated at 37 °C for 4 h. Supernatants were aspirated, and crystals were resolved by adding 500 µL dimethylsulfoxide (DMSO; ITW Reagents, Monza, Italy) per well and incubating overnight at room temperature while shaking. Supernatants were centrifuged for 6 min at 8000 rpm at room temperature and 100 µL were transferred to a 96-well plate (Sarstedt, Nümbrecht, Germany) in quadruplicates. The optical density was measured at 570 nm (OD_570_) against a blank (DMSO) using the Synergy HTX Multimode Reader (BioTek, Winooski, VT, USA) and normalized to 10,000 cells.

### Live/dead staining

hG-MSC cultures were stained using the Live-Dead Cell Staining Kit (Enzo Biochem, Farmingdale, NY, USA) according to the manufacturer’s protocol as previously described [36]. Briefly, gels were washed with PBS, stained with 150 µL staining solution containing Live-dye™ (excitation at 488 nm, emission at 518 nm) and propidium iodide (PI; excitation at 488 nm; emission at 615 nm), and incubated for 15 min at 37 °C. After washing once with PBS, samples were analyzed with the Revolve fluorescence microscope (ECHO, San Diego, CA, USA) using a 4x objective.

### RNA isolation

hG-MSCs were harvested using VitroGel^®^ Organoid Recovery Solution (TheWell Bioscience, North Brunswick Township, NJ, USA) according to the manufacturer’s protocol. The supernatant was collected and stored at -80 °C for ELISA (24–48 h), hydrogels were washed twice with Dulbecco’s Phosphate-Buffered Saline (DPBS, -Ca, -Mg; Gibco, Waltham, MA, USA), and hydrogels were transferred to 1.5 mL RNase free tubes (Sarstedt, Nümbrecht, Germany) using in total 600 µL of VitroGel^®^ Organoid Recovery Solution. Tubes were rocked and incubated for 2 min at 37 °C three times. Afterwards, the hydrogel was removed by centrifugation, and the cell pellet was washed with 200 µL DPBS. Cells were collected by centrifugation the supernatant was aspirated. Total RNA isolation was performed using the RNeasy Mini kit (Qiagen, Venlo, Netherlands) by adding 350 µL of RLT Lysis Buffer containing 2-mercaptoethanol (VWR, Radnor, PA, USA) and following the manufacturer’s protocol. RNA was eluted with 50 µL of RNase-free water, and RNA concentration was determined with the Synergy HTX Multimode Reader (BioTek, Winooski, VT, USA) using the Take3 and Take3 Trio Micro-Volume Plate (BioTek, Winooski, VT, USA).

### Reverse Transcription-Quantitative Polymerase Chain Reaction (RT-qPCR)

cDNA was generated from isolated RNA using the High-Capacity cDNA Reverse Transcription Kit (Applied Biosystems, Waltham, MA, USA) in accordance with the instructions provided by the manufacturer. Reverse transcription was performed with 3.5 ng/µL RNA as input material and the Biometra TOne PCR Thermal Cycler (Analytik Jena, Jena, Germany). Subsequent qPCR was performed according to the manufacturer’s instructions using TaqMan Gene Expression Master Mix (Applied Biosystems, Waltham, MA, USA) and following TaqMan gene expression assays (ThermoFisher Scientific, Waltham, MA, USA): IL-8*/CXCL8* (Hs00174103_m1), *IDO-1* (Hs00984146_m1), COX-2/*PTGS2* (Hs00153133_m1), PD-L1*/CD274* (Hs00204257_m1), PD-L2*/PDCD1LG2* (Hs00228839_m1), TSG-6*/TNFAIP6* (Hs00200180_m1), and *GAPDH* (Hs99999905_m1). Samples were processed using the QuantStudio 3 Real-Time PCR system (Applied Biosystems, Waltham, MA, USA) under the following conditions: initiation at 95 °C for 10 min, denaturation at 95 °C for 15 s, annealing and elongation at 60 °C for 1 min. Denaturation, annealing, and elongation phases were repeated for 50 cycles. Resulting C_t_-values were used to calculate relative changes in gene expression by using the formulas n-fold = 2 ^–ΔΔCt^ and ΔΔCt = (C_t_^target^ – C_t_^GAPDH^)_sample_ - (C_t_^target^ – C_t_^GAPDH^)_reference_, whereby *GAPDH* served as housekeeping gene. To analyze the effects of culture models on immune mediator expression, hG-MSCs cultured on TCP were used as the reference for each cytokine. To evaluate the responsiveness of hG-MSCs to pro-inflammatory cytokines, unstimulated (basal) hG-MSCs were used as the reference within each culture model. These normalization strategies enable (i) the evaluation of the effects of each culture model on immune mediator expression and (ii) the comparison of cytokine responsiveness across models to reveal, for example, diffusion-related differences between hydrogels and 2D cultures.

### Enzyme-Linked Immunosorbent Assay (ELISA)

Collected supernatants (0–24 h and 24–48 h) were centrifuged to remove any cell debris, aliquoted and stored at -80 °C until analysis. Quantification of interleukin-8 (IL-8) and PGE_2_ was performed using the Human IL-8 Uncoated ELISA (Invitrogen, Waltham, MA, USA) and the Prostaglandin E2 Parameter Assay (R&D Systems, Minneapolis, MN, USA), following the manufacturers’ instructions. The optical densities at 450 nm (OD_450_) and at 570 nm (OD_570_) were determined using the Synergy HTX Multimode Reader (BioTek, Winooski, VT, USA). Wavelength subtraction was performed by subtracting OD_570_ from OD_450_, and IL-8 and PGE_2_ concentrations were calculated based on the respective standard curve. The assay detection levels were 4-250 pg/mL and 39 − 2,500 pg/mL for IL-8 and PGE2 ELISAs, respectively. Values below the detection level were considered to be zero.

### Statistical analysis

In this study, experiments were conducted with six biological and one to four technical replicates. Statistical analysis and data visualization were performed using IBM SPSS Statistics 27.0.1.0 (IBM, Armonk, NY, USA) and GraphPad Prism 10.4.1 (Dotmatics, Boston, MA, USA), respectively. Statistical analysis of RT-qPCR data was performed using △△Ct values. Due to the low sample size (*n* = 6), the non-parametric Friedman ANOVA with subsequent Wilcoxon signed-rank *post-hoc* test was performed. *P*-values ≤ 0.05 were considered statistically significant.

## Results

### “VitroGel MSC^®^” shows low stiffness

“VitroGel^®^ MSC” started to polymerize 1 min after the addition of the media and ruptured after 57 min (Additional file 1A+ B). Dynamic mechanical analysis by cyclic compression determined a stiffness of 0.5 kPa at 10% compression (Additional file 1C). Moreover, after 10 compression cycles, the hydrogel exhibited no further reduction in thickness (Additional file 1D).

### Hydrogel embedding reduces hG-MSC metabolic activity, with viability loss upon IFN-γ and TNF-α stimulation

**Fig. 1:**
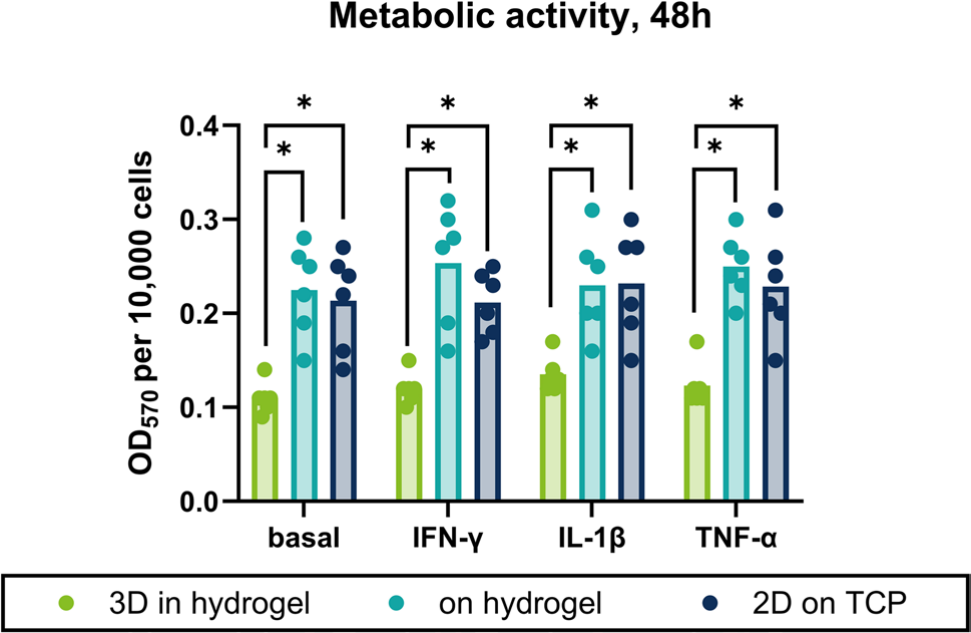
Metabolic activity of 3D-embedded hG-MSCs in resting and pro-inflammatory states. hG-MSCs isolated from six different donors (*n* = 6) were cultured in “VitroGel^®^ MSC” and stimulated with pro-inflammatory cytokines (100 ng/mL IFN-γ, 5 ng/mL IL-1β, 10 ng/mL TNF-α) for 48 h. Metabolic activity was quantified using the MTT assay, with the measured optical density (OD_570_) being normalized to 10,000 cells. hG-MSCs cultured on hydrogel and in 2D on tissue culture plastic (TCP) without hydrogel served as controls. Data are presented as means, with each data point within a group representing one donor. Statistical significance was assessed using by Friedman’s ANOVA with the Wilcoxon signed-rank test. * – statistically significant difference between indicated groups with *p* ≤ 0.05

In basal, as well as pro-inflammatory conditions, 3D-cultivation of hG-MSCs in hydrogel resulted in a more than 2-fold statistically significant reduction in metabolic activity compared to cultivation of hG-MSCs on hydrogel or TCP (Fig. 1). No significant difference was detected between hG-MSCs cultured on hydrogel and TCP. This suggests that hG-MSC metabolic activity may not be influenced by the hydrogel itself, but rather by the culture model.

**Fig. 2:**
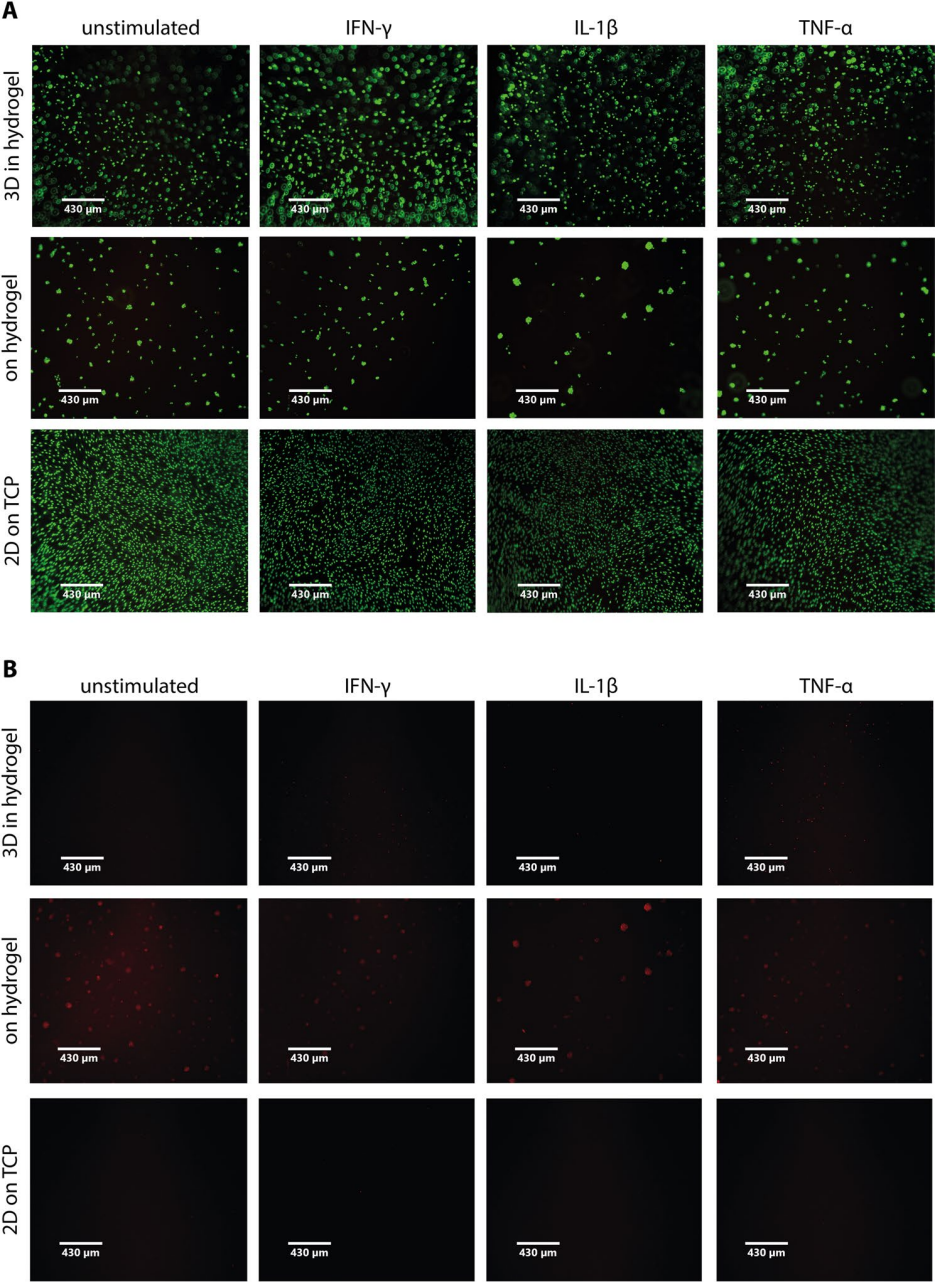
Representative images from a single donor showing 3D-embedded hG-MSC viability under resting and pro-inflammatory conditions. hG-MSCs were cultured in “VitroGel^®^ MSC” and stimulated with pro-inflammatory cytokines (100 ng/mL IFN-γ, 5 ng/mL IL-1β, 10 ng/mL TNF-α) for 48 h. hG-MSCs cultured on hydrogel and in 2D on tissue culture plastic (TCP) without hydrogel served as controls. Viability of hG-MSCs was visualized by fluorescence microscopy using green Live-Dye^™^ (**A**) and red PI stain (**A** + **B**). Pictures were taken with the ECHO Revolve fluorescence microscope using the 4-fold objective

The majority of hG-MSCs cultured in hydrogel or on TCP appear viable under both resting and pro-inflammatory conditions (Fig. 2A). However, 3D-cultured hG-MSCs in hydrogel exposed to pro-inflammatory cytokines, especially IFN-γ and TNF-α, exhibit a slightly higher number of dead cells compared to unstimulated hG-MSCs (Fig. 2B). In addition, hG-MSCs cultured on hydrogel appear not to attach properly to the hydrogel, forming spheroid-like structures with dead cells inside, under all conditions.

### Embedding hG-MSCs in hydrogel triggers pro-inflammatory IL-8 expression

**Fig. 3:**
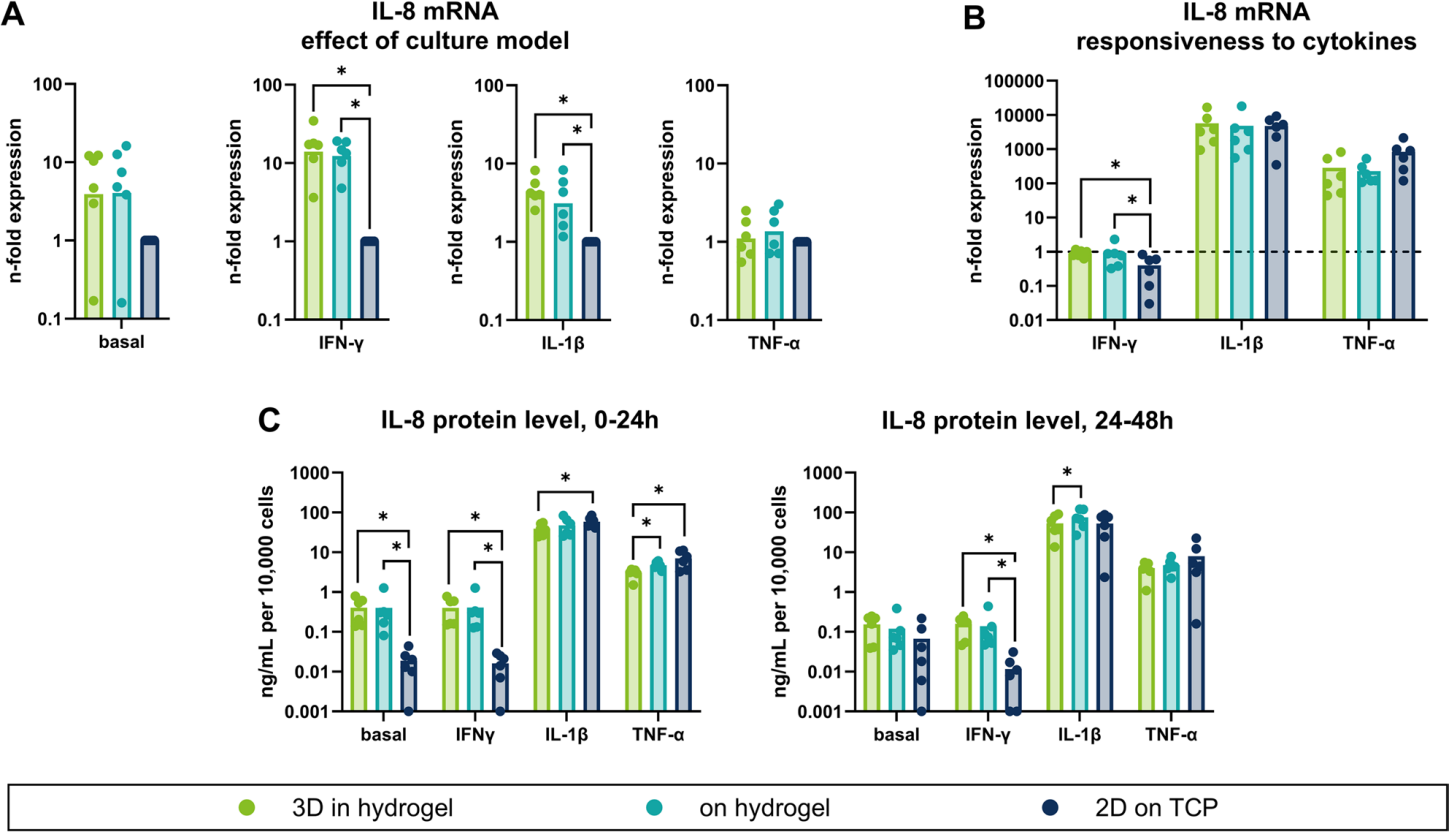
IL-8 response of 3D-embedded hG-MSCs under resting and pro-inflammatory conditions. hG-MSCs isolated from six different donors (*n* = 6) were cultured in “VitroGel^®^ MSC” and stimulated with pro-inflammatory cytokines (100 ng/mL IFN-γ, 5 ng/mL IL-1β, 10 ng/mL TNF-α) for 48 h. hG-MSCs cultured on hydrogel and in 2D on tissue culture plastic (TCP) without hydrogel served as controls. IL-8 mRNA expression was quantified by qPCR. To analyze the effect of the culture models on IL-8 mRNA expression, results were normalized to IL-8 expression of hG-MSCs cultured in 2D on TCP for each cytokine respectively (**A**). The reactivity of hG-MSCs to pro-inflammatory cytokines was quantified by normalizing the results to the basal IL-8 mRNA expression of each culture model, as indicated by the dashed line (**B**). IL-8 protein levels from 0–24 h and 24–48 h after initiating pro-inflammatory stimulation were quantified by ELISA (**C**). Data are presented as means, with each data point within a group representing one donor. Statistical significance was assessed using Friedman’s ANOVA with the Wilcoxon signed-rank test. * – statistically significant difference between indicated groups with *p* ≤ 0.05

When observing the effect of the culture model (Fig. 3A), IL-8 mRNA expression was elevated in 3D-embedded hG-MSCs and hG-MSCs cultured on hydrogel compared to those cultured on TCP under basal (3.9- and 4.1-fold change, respectively), IFN-γ (14.0- and 12.3-fold change, respectively), and IL-1β (4.4- and 3.1-fold change, respectively) conditions, with statistical significance observed for IFN-γ and IL-1β groups. In contrast, no significant differences in IL-8 mRNA expression were detected upon TNF-α stimulation in hG-MSCs 3D-embedded in hydrogel or cultured on hydrogel (1.1- and 1.4-fold change, respectively) compared to hG-MSCs cultured on TCP.

Analysis of hG-MSC responsiveness to cytokines (Fig. 3B) revealed significantly higher IL-8 mRNA expression upon IFN-γ stimulation in 3D-embedded hG-MSCs and hG-MSCs cultured on hydrogel compared to conventional 2D cultures on TCP (0.9-, 0.7-, 0.2-fold change). No significant difference in the responsiveness of hG-MSCs to IL-1β (3688-, 2495-, 3267-fold change) and TNF-α (158-, 189-, 564-fold change) was found between different culture models. However, a tendency of hG-MSCs cultured on TCP showing increased IL-8 mRNA expression than the other culture models, was observed under TNF-α-stimulation.

When analyzing differences in IL-8 protein response between culture models (Fig. 3C), significantly elevated IL-8 levels were detected after 0–24 h of culture for 3D-embedded hG-MSCs and hG-MSCs cultured on hydrogel compared to hG-MSCs cultured on TCP under basal (53.9, 53.7, 2.5 pg/mL) and IFN-γ (53.7, 54.6, 2.1 pg/mL) conditions. In contrast, IL-8 protein levels were significantly decreased for 3D-cultured hG-MSCs compared to hG-MSCs cultured on TCP upon IL-1β- (5210 and 7800 pg/mL), and TNF-α-stimulation (400 and 942 pg/mL). Additionally, 3D-embedded hG-MSCs exhibited lower IL-8 protein levels than hG-MSCs cultured on hydrogel (IL-1β: 5210 and 6312 pg/mL; TNF-α: 400 and 626 pg/mL), with statistical significance in the TNF-α-stimulated group. After 24–48 h of culture, a similar trend was visible. IL-8 protein levels were elevated for basal and IFN-γ-stimulated hG-MSCs 3D-embedded in hydrogel and hG-MSCs cultured on hydrogel compared to hG-MSCs cultured on TCP (basal: 20.6, 15.9, 8.9 pg/mL; IFN-γ: 21.3, 18.6, 1.5 pg/mL), with statistical significances for IFN-γ group. Upon IL-1β-stimulation, hG-MSCs 3D-embedded in hydrogel still demonstrated significantly lower IL-8 protein levels than hG-MSCs cultured on hydrogel, but similar levels to hG-MSCs cultured on TCP (7112, 10057, 7020 pg/mL). Under TNF-α stimulation, IL-8 protein levels were comparable between hG-MSCs 3D-embedded in hydrogel and hG-MSCs cultured on hydrogel, with a tendency of slightly higher levels in hG-MSCs cultured on TCP (536, 627, 1062 pg/mL).

### Exposing hG-MSCs to hydrogel increases COX-2 and PGE_2_ expression

**Fig. 4:**
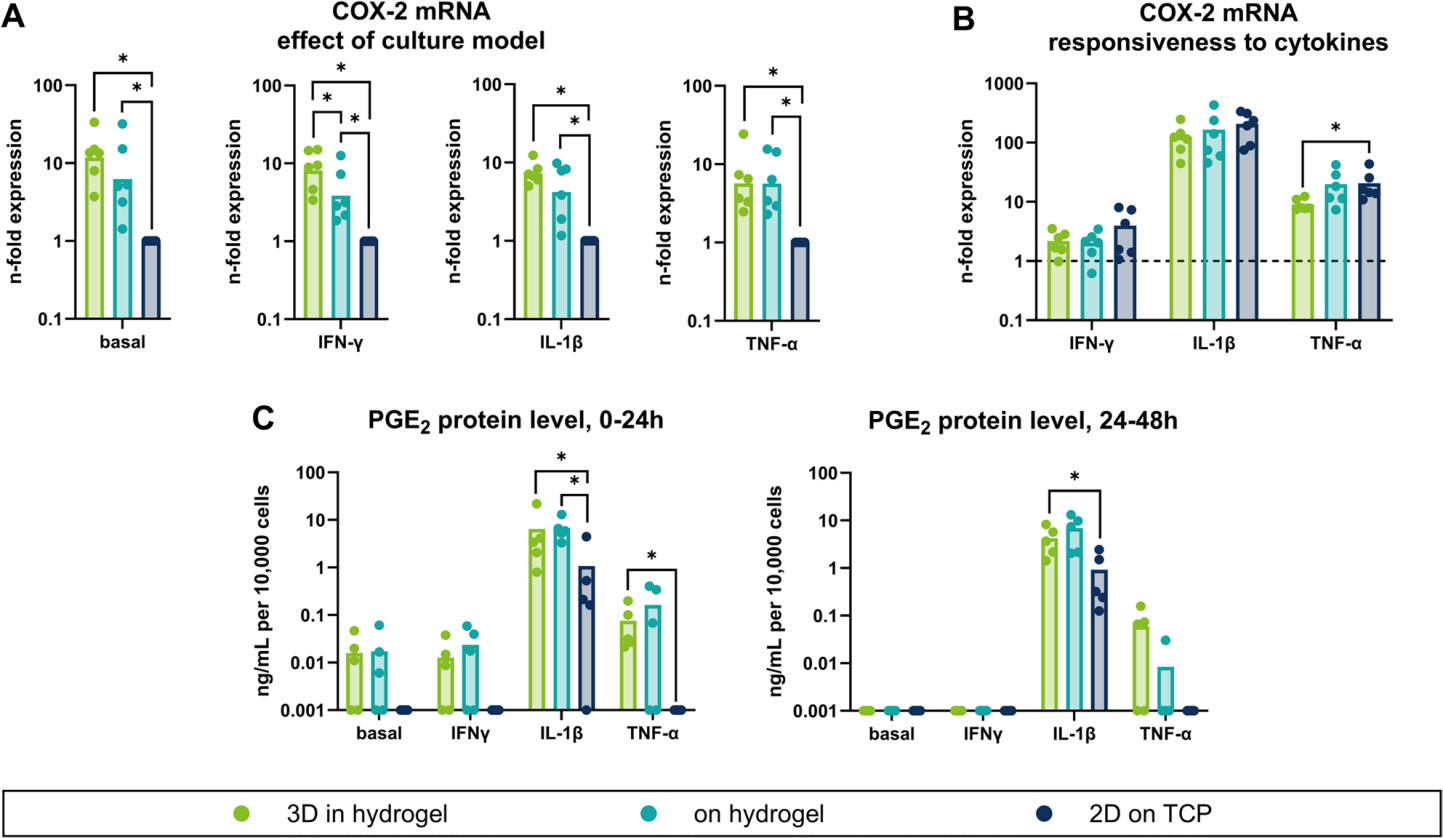
COX-2 and PGE_2_ response of 3D-embedded hG-MSCs under resting and pro-inflammatory conditions. hG-MSCs isolated from six different donors (*n* = 6) were cultured in “VitroGel^®^ MSC” and stimulated with pro-inflammatory cytokines (100 ng/mL IFN-γ, 5 ng/mL IL-1β, 10 ng/mL TNF-α) for 48 h. hG-MSCs cultured on hydrogel and in 2D on tissue culture plastic (TCP) without hydrogel served as controls. COX-2 mRNA expression was quantified by qPCR. To analyze the effect of the culture models on COX-2 mRNA expression, results were normalized to COX-2 mRNA expression of hG-MSCs cultured in 2D on TCP for each cytokine respectively (**A**). The reactivity of hG-MSCs to pro-inflammatory cytokines was quantified by normalizing the results to the basal COX-2 mRNA expression of each culture model, as indicated by the dashed line (**B**). PGE_2_ protein levels from 0–24 h and 24–48 h after initiating pro-inflammatory stimulation were quantified by ELISA (**C**). Data are presented as means, with each data point within a group representing one donor. Statistical significance was assessed using Friedman’s ANOVA with the Wilcoxon signed-rank test. * – statistically significant difference between indicated groups with *p* ≤ 0.05

When analyzing the response to the culture model (Fig. 4A), COX-2 mRNA expression was significantly higher in 3D-embedded hG-MSCs and hG-MSCs cultured on hydrogel compared to hG-MSCs maintained on TCP. This increase was observed under basal conditions (11.8- and 6.2-fold change, respectively) and after stimulation with the pro-inflammatory cytokines IFN-γ (8.1- and 3.9-fold change, respectively), IL-1β (7.3- and 4.2-fold change, respectively), and TNF-α (5.7- and 5.7-fold change, respectively). Furthermore, COX-2 mRNA expression was higher in 3D-embeddded hG-MSCs compared to hG-MSCs cultured on hydrogel under basal, IFN-γ and IL-1β conditions, reaching statistical significance in the IFN-γ group. Upon TNF-α stimulation, no difference was detected between 3D-embedded hG-MSCs and hG-MSCs cultured on hydrogel.

Analysis of hG-MSC responsiveness to cytokines (Fig. 4B) revealed slight tendencies of lower COX-2 response in 3D-embedded hG-MSCs and hG-MSCs cultured on hydrogel compared to hG-MSCs cultured on TCP in response to IFN-γ- (2.0-, 1.8-, 2.9-fold change) and IL-1β (109.5-, 119.5-, 177.7-fold change). 3D-embedded hG-MSCs showed lower TNF-α-induced COX-2 mRNA expression than hG-MSCs cultured on hydrogel or on TCP (8,9-, 16.7-, 18.4-fold change), but significant differences were detected only for hG-MSCs cultured on TCP.

When analyzing differences in PGE_2_ protein response between culture models (Fig. 4C), elevated PGE_2_ levels were observed between 0 and 24 h for 3D-embedded hG-MSCs and hG-MSCs cultured on hydrogel compared to hG-MSCs cultured on TCP in basal state (15.5, 16.7, 0 pg/mL) and after IFN-γ stimulation (12.3, 23.3, 0 pg/mL). Following IL-1β stimulation, PGE_2_ levels were significantly elevated by hG-MSCs 3D-embedded in hydrogel, or cultured on hydrogel compared to hG-MSCs cultured on TCP (6434, 6810, 1071 pg/mL). TNF-α stimulation resulted in higher PGE_2_ levels for 3D-embedded hG-MSCs and hG-MSCs cultured on hydrogel than hG-MSCs on TCP (75.7, 162.6, 0 pg/mL), with a statistical significance observed for 3D-embedded hG-MSCs. No significant differences were detected between hG-MSCs 3D-embeddded in hydrogel and hG-MSCs cultured on hydrogel under any of the culture conditions or pro-inflammatory environments, however tendencies of hG-MSCs cultured on hydrogel showing increased PGE_2_ levels were visible. PGE_2_ levels of basal, IFN-γ-, and TNF-α-stimulated hG-MSCs cultured on TCP could not be detected. After 24–48 h of culturing, PGE_2_ could no longer be detected in the basal state or under IFN-γ stimulation in hG-MSCs from any of the culture models. Upon IL-1β stimulation, 3D-embedded hG-MSCs still showed significantly higher PGE_2_ levels than hG-MSCs on TCP, and a tendency of lower PGE_2_ levels than hG-MSCs on hydrogel (4237, 6887, 923 pg/mL). When stimulated with TNF-α, the PGE_2_ levels of hG-MSCs 3D-embedded in hydrogel remained elevated, whereas PGE_2_ levels of hG-MSCs on hydrogel decreased, and those of hG-MSCs on TCP could still not be detected (59.5, 7.6, 0 pg/mL).

### Hydrogel-embedded hG-MSCs display elevated TSG-6 expression, whereas the alterations in IDO-1 expression depend on the inflammatory environment

**Fig. 5:**
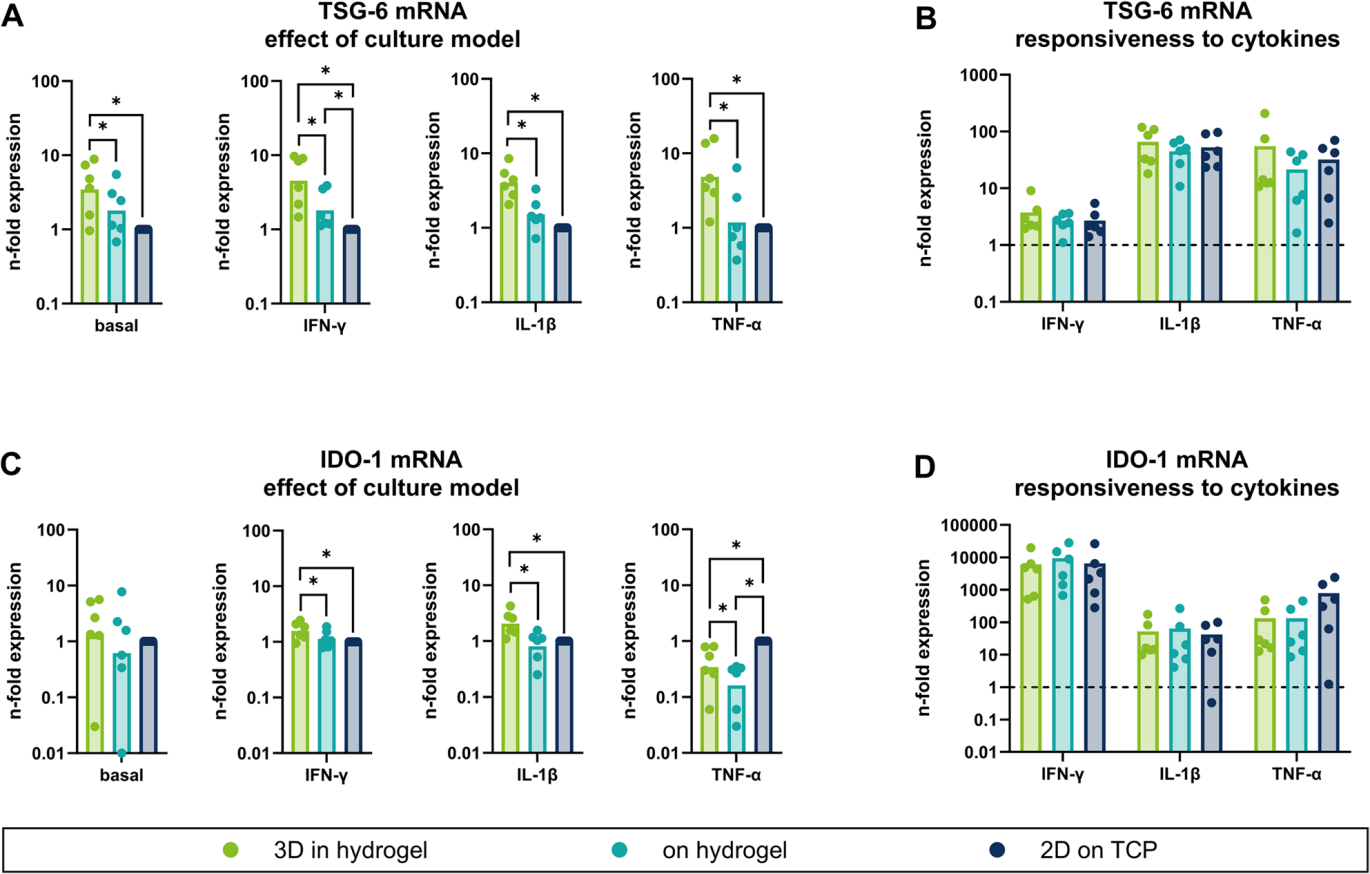
TSG-6 and IDO-1 response of 3D-embedded hG-MSCs under resting and pro-inflammatory conditions. hG-MSCs isolated from six different donors (*n* = 6) were cultured in “VitroGel^®^ MSC” and stimulated with pro-inflammatory cytokines (100 ng/mL IFN-γ, 5 ng/mL IL-1β, 10 ng/mL TNF-α) for 48 h. hG-MSCs cultured on hydrogel and in 2D on tissue culture plastic (TCP) without hydrogel served as controls. TSG-6 (**A**, **B**) and IDO-1 (**C**, **D**) mRNA expression was quantified by qPCR. To analyze the effect of the culture models on TSG-6 and IDO-1 mRNA expression, results were normalized to the immune mediator expression of hG-MSCs cultured in 2D on TCP for each cytokine respectively (**A**, **C**). The reactivity of hG-MSCs to pro-inflammatory cytokines was quantified by normalizing results to the basal expression of immune mediators in each culture model, as indicated by the dashed lines (**B**, **D**). Data are presented as means, with each data point within a group representing one donor. Statistical significance was assessed using Friedman’s ANOVA with the Wilcoxon signed-rank test. * – statistically significant difference between indicated groups with *p* ≤ 0.05

When analyzing the response to the culture model (Fig. 5A), TSG-6 mRNA expression was significantly higher in 3D-embedded hG-MSCs than in hG-MSCs cultured on hydrogel or on TCP under basal (3.5- and 1.8-fold change, respectively), as well as pro-inflammatory conditions (IFN-γ: 4.5- and 1.8-fold change; IL-1β: 4.0- and 1.5-fold change; TNF-α: 4.8- and 1.2-fold change, respectively). Moreover, hG-MSCs cultured on hydrogel showed a tendency to express higher levels of TSG-6 mRNA than hG-MSCs on TCP, with a statistical significance observed for IFN-γ conditions.

Analysis of hG-MSC responsiveness to cytokines (Fig. 5B) revealed no significant differences in TSG-6 mRNA expression between culture models. However, a tendency of 3D-embedded hG-MSCs showing increased TSG-6 mRNA response compared to hG-MSCs cultured on hydrogel or on TCP in all pro-inflammatory conditions (IFN-γ: 3.2-, 2.5-, 3.4-fold change; IL-1β: 52.0-, 38.1-, 44.8-fold change, TNF-α: 26.7-, 12.6-, 19.2-fold change) was noted.

The IDO-1 mRNA expression in response to the culture model (Fig. 5C) was elevated when hG-MSCs were 3D-embedded in hydrogel compared to those maintained on hydrogel, under basal (1.2- and 0.6-fold change), as well as pro-inflammatory conditions (IFN-γ: 1.6- and 1.1-fold change; IL-1β: 2.1- and 0.8-fold change; TNF-α: 0.3- and 0.2-fold change), with statistical significances for cytokine treated groups. Moreover, hG-MSCs 3D-embedded in hydrogel exhibited a significantly higher IDO-1 mRNA expression than hG-MSCs cultured on TCP when stimulated with IFN-γ (1.6-fold change), or IL-1β (2.1-fold change), but a significantly lower IDO-1 mRNA expression, when stimulated with TNF-α (0.3-fold change). Basal 3D-embedded hG-MSCs showed a tendency to express slightly more IDO-1 mRNA compared to hG-MSCs cultured on TCP (1.3-fold change). hG-MSCs tended to lower their expression of IDO-1 mRNA, when cultured on hydrogel compared to when cultured on TCP in basal (0.6-fold change), IL-1β (0.8-fold change) or TNF-α (0.2-fold change; *p* ≤ 0.05) conditions.

Analysis of hG-MSC responsiveness to pro-inflammatory cytokines (Fig. 5D) revealed no significant differences in IDO-1 mRNA expression between all culture models. However, 3D-embedded hG-MSCs tended to exhibit higher IDO-1 mRNA expression than hG-MSCs cultured on TCP in response to IFN-γ (3091- and 2543-fold change) and IL-1β (28- and 17-fold change), but lower expression in response to TNF-α (49- and 187-fold change). Moreover, 3D-embedded G-MSCs displayed lower IDO-1 expression than hG-MSCs cultured on hydrogel in response to IFN-γ (3091- and 4627-fold change), higher expression in response to IL-1β (28- and 23-fold change), and comparable expression in response to TNF-α (49- and 48-fold change).

### Hydrogel-embedded hG-MSCs display altered PD-L1 and PD-L2 expression depending on the inflammatory environment

**Fig. 6:**
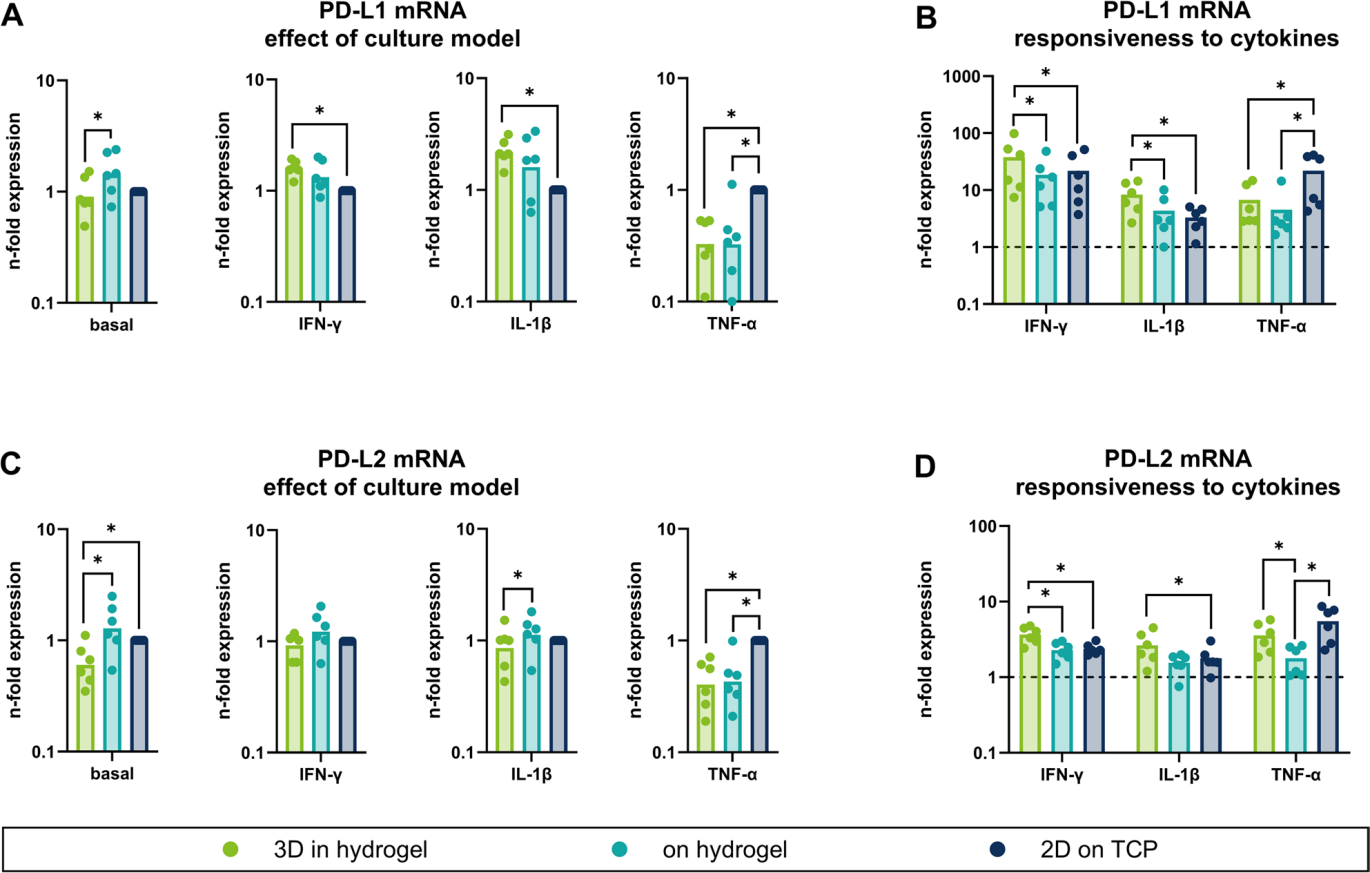
PD-L1 and PD-L2 response of 3D-embedded hG-MSCs under resting and pro-inflammatory conditions. hG-MSCs isolated from six different donors (*n* = 6) were cultured in “VitroGel^®^ MSC” and stimulated with pro-inflammatory cytokines (100 ng/mL IFN-γ, 5 ng/mL IL-1β, 10 ng/mL TNF-α) for 48 h. hG-MSCs cultured on hydrogel and in 2D on tissue culture plastic (TCP) without hydrogel served as controls. PD-L1 (**A**, **B**) and PD-L2 (**C**, **D**) mRNA expression was quantified by qPCR. To analyze the effect of the culture models on PD-L1 and PD-L2 mRNA expression, results were normalized to the immune mediator expression of hG-MSCs cultured in 2D on TCP for each cytokine respectively (**A**, **C**). The reactivity of hG-MSCs to pro-inflammatory cytokines was quantified by normalizing results to the basal expression of immune mediators in each culture model, as indicated by the dashed lines (**B**, **D**). Data are presented as means, with each data point within a group representing one donor. Statistical significance was assessed using Friedman’s ANOVA with the Wilcoxon signed-rank test. * – statistically significant difference between indicated groups with *p *≤ 0.05

Analysis of PD-L1 response to the culture model (Fig. 6A) revealed under basal conditions a significantly lower PD-L1 mRNA expression in 3D-embedded hG-MSCs than in hG-MSCs cultured on hydrogel (0.9- and 1.4-fold change). Furthermore, hG-MSCs 3D-embedded in hydrogel exhibited a slight and non-significant decrease in PD-L1 mRNA expression compared to hG-MSCs on TCP (0.9-fold change). Following stimulation with IFN-γ or IL-1β, hG-MSCs 3D-embedded in hydrogel exhibited increased PD-L1 mRNA expression levels compared to hG-MSCs on hydrogel and hG-MSCs on TCP with a statistical significance for the latter group (IFN-γ: 1.6- and 1.3-fold change; IL-1β: 2.2- and 1.6-fold change). No significant differences were detected between hG-MSCs on hydrogel and hG-MSCs on TCP. When stimulated with TNF-α, PD-L1 mRNA expression decreased significantly in hG-MSCs 3D-embedded in hydrogel and hG-MSCs cultured on hydrogel compared to those cultured on TCP (0.3- and 0.3-fold change). No differences in PD-L1 mRNA expression were detected between hG-MSCs 3D-embedded in hydrogel and hG-MSCs cultured on hydrogel.

When analyzing hG-MSC responsiveness to cytokines (Fig. 6B), 3D-embedded hG-MSCs exhibited a significantly higher PD-L1 mRNA expression than hG-MSCs cultured on hydrogel or on TCP in response to IFN-γ (25.5-, 13.5-, 14.5-fold change) and IL-1β (7.1-, 3.3-, 2.9-fold change). In contrast, upon TNF-α stimulation, hG-MSCs 3D-embedded in hydrogel and hG-MSCs cultured on hydrogel showed significantly lower PD-L1 mRNA response than hG-MSCs cultured on TCP (5.3-, 3.3-, 14.6-fold change).

A similar response to culture model was observed for PD-L2 mRNA expression (Fig. 6C) under basal and TNF-α conditions. Basal 3D-embedded hG-MSCs exhibited significantly lower PD-L2 mRNA expression than those cultured on hydrogel or on TCP (0.6- and 1.3-fold change). Higher PD-L2 mRNA levels were detected in hG-MSCs on hydrogel than in hG-MSCs on TCP, but without statistical significance. Upon IFN-γ stimulation, PD-L2 mRNA expression did not differ significantly among culture models. Yet, 3D-embedded hG-MSCs tended to express lower, and hG-MSCs cultured on hydrogel tended to express slightly higher PD-L2 mRNA levels than hG-MSCs cultured on TCP (0.9- and 1.2-fold change). IL-1β-stimulated 3D-embedded hG-MSCs exhibited significantly decreased PD-L2 mRNA expression than hG-MSCs cultured on hydrogel and slightly lower levels than those cultured on TCP (0.9- and 1.1-fold change). No significant difference was detected between hG-MSCs cultured on hydrogel and hG-MSCs on TCP. Upon TNF-α stimulation, hG-MSCs exhibited significantly decreased PD-L2 mRNA expression when 3D-embedded in hydrogel or cultured on hydrogel, compared to hG-MSCs cultured on TCP (0.4- and 0.4-fold change). No differences in PD-L2 mRNA expression were detected between 3D-embedded hG-MSCs and hG-MSCs cultured on hydrogel.

Similar to PD-L1 mRNA response, analysis of hG-MSC responsiveness to cytokines (Fig. 6D) revealed significantly increased PD-L2 mRNA expression in 3D-embedded hG-MSCs compared to hG-MSCs cultured on hydrogel and on TCP upon IFN-γ- (3.6-, 2.2-, 2.3-fold change) and IL-1β-stimulation (2.4-, 1.5-, 1.7-fold change). In contrast, TNF-α stimulation led to a decreased PD-L2 mRNA expression in 3D-embedded hG-MSCs and hG-MSCs cultured on hydrogel compared to hG-MSCs on TCP (3.3-, 1.6-, 4.9-fold change). Still, 3D-embedded hG-MSCs showed significantly higher responsiveness in form of PD-L2 expression than hG-MSCs cultured on hydrogel.

## Discussion

The present study evaluated how a 3D microenvironment, created by embedding hG-MSCs in hydrogel, affects cellular viability, metabolic activity, and immune mediator expression under basal and pro-inflammatory conditions (IFN-γ, IL-1β, or TNF-α), compared to conventional 2D cultures. Our findings demonstrate that hydrogel embedding alters key cellular responses in hG-MSCs, underscoring the critical role of the 3D microenvironment in regulating MSC behavior.

Embedding hG-MSCs in hydrogel consistently reduced metabolic activity by approximately twofold across all conditions compared to cells cultured on hydrogel or in conventional 2D culture on TCP. This finding indicates that the 3D microenvironment itself, rather than pro-inflammatory stimulation, mainly influences metabolic activity. These findings are consistent with Pangjantuk et al., who reported reduced metabolic activity in human umbilical cord-derived MSCs (hUC-MSCs) embedded in alginate-hyaluronic acid hydrogels after 3 days [37]. Similarly, Moshaverinia et al. demonstrated reduced metabolic activity in MSCs encapsulated in larger (> 500 μm) alginate hydrogel microbeads compared to smaller (427 μm) RGD-coupled alginate hydrogel microbeads [38]. These observations suggest that limited diffusion and nutrient transport within 3D hydrogels, rather than inflammatory cues, contribute to reduced metabolic activity. Beyond diffusion constraints, hydrogel composition [39] and internal cell migration [40] may also affect assay readouts. In contrast, these confounding factors are largely negligible in 2D cultures due to direct cell exposure to culture media [39], the absence of a hydrogel, and reduced planar migration. Hence, direct comparisons of metabolic activity between 2D and 3D models must be interpreted with caution [41].

While metabolic activity was reduced, cell viability remained largely preserved in hydrogel-embedded hG-MSCs compared to conventional 2D cultures. This might indicate that hydrogel embedding induces a quiescent metabolic state that suppresses mitochondrial function, without affecting membrane integrity. Moreover, a slightly elevated number of dead cells was observed upon IFN-γ- or TNF-α-stimulation, a finding not previously reported in the literature. Because 2D-cultured cells did not exhibit this effect, we hypothesize that the 3D microenvironment sensitizes hG-MSCs to these cytokines. Moreover, hG-MSCs cultured on top of hydrogel formed spheroid-like structures with apoptotic cores, a phenomenon already observed by Nguyen et al., who reported apoptotic cores after culturing spheroids of 100 and 500 cells for 10 days [42]. This effect may be attributed to the low stiffness (500 Pa) of the hydrogel, which mimics non-adherent substrates such as agarose or hyaluronic acid commonly used for spheroid production [43], leading to insufficient cell attachment, the formation of aggregates, limited nutrient diffusion, and ultimately apoptosis.

To further investigate the immunomodulatory response of hG-MSCs to the transition from 2D to 3D culture conditions, we analyzed the expression of key immune mediators involved in tissue remodeling and immune regulation [5]. Therefore, we focused on representative mediators, including IL-8, COX-2, PGE_2_, TSG-6, IDO-1, PD-L1, and PD-L2.

One of the key findings of this study is the significantly elevated IL-8 expression in hG-MSCs embedded within or cultured on top of hydrogels, observed at both mRNA and protein levels. This increase was most pronounced under basal and IFN-γ-stimulated conditions. Since spheroid formation occurred in the on-top cultures, it remains unclear whether this IL-8 upregulation is primarily driven by the hydrogel material itself or by spheroid-induced 3D cell-cell interactions, given that spheroids are known to secrete higher levels of soluble factors [44], including IL-8 [45]. Comparable findings were reported by Yao et al., who observed a 28-fold IL-8 mRNA increase in adipose tissue-derived MSCs encapsulated in gelatin/sodium alginate hydrogel microspheres versus 2D cultures [46]. Also Zhu et al. demonstrated a significantly higher IL-8 production in hUC-MSCs cultured in 3D using a chitin-based hydrogel [47]. Collectively, these findings suggest that 3D microenvironments prime hG-MSCs toward a pro-inflammatory, secretory phenotype by inducing IL-8 expression, potentially initiating downstream inflammatory signaling cascades.

Hydrogel embedding also significantly enhanced COX-2 mRNA expression and PGE_2_ secretion in hG-MSCs under both basal and cytokine-stimulated conditions. This finding aligns with Gonzalez-Pujana et al., who demonstrated increased PTGS2 mRNA expression in human bone marrow MSCs three days after priming with IFN-γ and TNF-α and embedding in alginate-collagen hydrogels [48]. Elevated COX-2 and PGE_2_ levels in spheroid-like structures in on-top cultures are consistent with previous reports [49–51] linking spheroid architecture to increased paracrine activities [44]. The slightly higher COX-2 mRNA expression in hydrogel-embedded hG-MSCs compared to on-top cultures implies that 3D-embedding has a more pronounced immunomodulatory effect than spheroid-like structures, underscoring the influence of the 3D environment created by hydrogel-embedding.

Similarly, hydrogel encapsulation markedly upregulated TSG-6 mRNA expression under all conditions, compared to on-top cultured hG-MSCs, and conventional 2D cultures. This partially aligns with the findings of Kojima et al.., who reported significantly higher TSG-6 expression in 3D-hydrogel embedded hG-MSCs than 2D-cultures upon TNF-α exposure, but no difference between these groups in basal state [52]. The discrepancy to our findings could be explained by differences in hydrogel stiffness. The decellularized extracellular matrix of liver used by Kojima et al. exhibits a storage modulus of approximately 15 kPa. In contrast, the hydrogel employed in our paper was softer (500 Pa), which may alter paracrine signaling [53, 54] and stress-related gene expression through altered cytoskeletal tension and yes-associated protein (YAP) nuclear translocation [55].

IDO-1 expression was also elevated in hydrogel-embedded hG-MSCs under basal, IFN-γ, and IL-1β conditions, but was notably suppressed by TNF-α. This is consistent with Gonzales-Pujana et al., who reported a similar increase in IDO-1 mRNA expression 3 days after IFN-γ and TNF-α-primed human bone marrow MSCs were embedded in alginate-collagen hydrogels [48]. Notably, Darnell et al. demonstrated that substrate stiffness exerts a greater influence on IDO-1 expression than cytokine stimulation, with stiffer matrices inducing stronger IDO-1 expression. In their experiment, mouse MSCs were encapsulated in alginate hydrogels of varying stiffness and stimulated either with or without a mixture of TNF-α and IFN-γ [56]. Although we did not test the influence of substrate stiffness, we found that cytokines do have a strong influence on IDO-1 expression in 3D-embedded hG-MSCs. In our study, IFN-γ was the most potent inducer of IDO-1 in 3D-embeded hG-MSCs, followed by IL-1β and TNF-α, suggesting that cytokine responsiveness is preserved, but modulated by the 3D microenvironment.

PD-L1 and PD-L2 expression patterns were highly condition-dependent. TNF-α suppressed both molecules in hydrogel-exposed hG-MSCs. In basal conditions, PD-L1 and PD-L2 were downregulated in 3D-embedded hG-MSCS, but elevated in on-top cultures, compared to 2D cultures. Under IFN-γ and IL-1β stimulation, PD-L1 expression increased significantly in hydrogel-exposed hG-MSCs. To our knowledge, this is the first study directly comparing PD-L1 and PD-L2 expression between hydrogel-embedded and conventionally 2D cultured hG-MSCs, highlighting the modulatory capacity of the 3D microenvironment on checkpoint molecule expression.

Across all environmental conditions, hG-MSCs exhibited cytokine-specific responses. IL-1β induced the strongest upregulation of IL-8 and COX-2 mRNA expression, followed by TNF-α, and IFN-γ. Notably, IL-8 expression remained unchanged upon IFN-γ stimulation in 2D cultures compared to the basal state, suggesting a possible inhibitory influence of TCP on IL-8 expression. Highest TSG-6 response was noted in hG-MSCs treated with IL-1β- or TNF-α, followed by IFN-γ. In contrast, IDO-1 mRNA expression was most strongly induced by IFN-γ, followed by TNF-α, and IL-1β. Similarly, PD-L1 mRNA expression was highest upon IFN-γ stimulation, followed by IL-1β and TNF-α. Contrary, no trend towards a specific pro-inflammatory cytokine increasing PD-L2 mRNA expression, was visible. Still, all cytokines led to an increased PD-L2 expression compared to the basal state in all culture models. These findings are consistent with previous reports describing distinct immune mediator expression patters in response to pro-inflammatory stimulation [6], reflecting regulation by cytokine-specific signaling pathways. IFN-γ is known to potently induce IDO-1, PD-L1, and PD-L2 via the JAK-STAT/IRF pathway [57], whereas IL-1β and TNF-α preferentially stimulate IL-8, COX-2, and TSG-6 expression through NF-κB and MAPK signaling [58, 59]. In addition, the 3D hydrogel microenvironment may modulate these responses via YAP/TAZ-mediated mechanotransduction [55, 60, 61] and HIF-1α-dependent hypoxia signaling [62, 63], thereby enhancing the expression of immunosuppressive mediators.

Pro-inflammatory cytokines were able to diffuse into the hydrogel and trigger immune mediator expression similar to 2D cultures. However, the magnitude of these responses differed between culture models, highlighting the strong influence of physical microenvironment on MSC behavior. Specifically, hydrogel embedding enhanced IL-8 responses to IFN-γ, as well as PD-L1 and PD-L2 responses to IFN-γ and IL-1β. In contrast, TNF-α stimulation reduced COX-2, PD-L1, and PD-L2 responses in hydrogel-embedded hG-MSCs compared to conventional 2D cultures. No significant differences in responsiveness were obtained regarding TSG-6 and IDO-1 mRNA expression. Collectively, these results demonstrate that both, the cytokine milieu and the culture model critically influence hG-MSC behavior and should therefore be carefully considered for MSC-based therapeutic strategies.

Taken together, hydrogel embedding profoundly alters the expression of mediators involved in the inflammatory and immunomodulatory functions in hG-MSCs, which might be associated with the transition from 2D to 3D conditions. Moreover, several factors may contribute to alterations in hG-MSC responses. One possible factor is impaired gas exchange within the hydrogel matrix, which can lead to hypoxia and low cellular stress, thereby triggering downstream pathways that promote the transcription of anti-inflammatory factors [64, 65]. Assessing hypoxia and related signaling pathways in hydrogel-embedded hG-MSCs could provide valuable insights into this mechanism. Moreover, substrate stiffness highly influences cell behavior. Softer matrices have been shown to enhance the secretion of anti-inflammatory immune mediators [53], primarily through mechanotransduction mechanisms involving the yes-associated protein/transcriptional co-activator with PDZ-binding motif (YAP-TAZ) pathway [55]. In addition, hydrogel porosity significantly influences nutrient diffusion, cell-matrix interactions, cell proliferation [66], and overall cell behavior, further modulating MSC paracrine activity [67]. Overall, priming MSCs and embedding them in soft hydrogels appears to potentiate their anti-inflammatory and immunomodulatory phenotype, which may ultimately enhance therapeutic efficacy in clinical applications.

### Study limitations and future perspectives

One limitation of our study is its focus on early time points (48 h after priming for mRNA levels; 0–24 h and 24–48 h after priming for protein levels) to capture the immediate immunomodulatory response of hG-MSCs to pro-inflammatory stimuli. This period is particularly relevant for clinical applications, as rapid immune regulation is critical. Later time points were not investigated as the aim was to characterize this fast, dynamic response phase rather than long-term adaptation.

Another limitation is the absence of a true 2D control on the hydrogel. Due to the material’s low stiffness, hG-MSCs did not form a monolayer, but instead formed spheroid-like aggregates resembling 3D cultures. Nevertheless, we included this condition as a material control to assess potential hydrogel-related effects on hG-MSC behavior. Overall, our data show that, compared to hG-MSCs cultured on top of hydrogels and conventional 2D cultures commonly used in research, embedding hG-MSCs in the soft “VitroGel^®^ MSC” hydrogel enhances expression of specific anti-inflammatory immune mediators. These findings suggest that 3D environments induced by hydrogel embedding may enhance the immunomodulatory capacity of hG-MSCs, thereby representing a promising approach for optimizing MSC-based therapies.

Future in vitro studies should focus on the time-dependent immune mediator profiles of 3D and 2D hG-MSC cultures at various time points in order to evaluate the adaptation, stability, and potential exhaustion of the immunomodulatory phenotype. Furthermore, key functional assays such as co-culture experiments with immune cells are required to verify whether the observed changes in immune mediator profiles also lead to altered immunomodulatory functions. Additionally, the properties of “VitroGel^®^ MSC”, such as porosity and degradability, and their effects on cell morphology and cytoskeletal remodeling should be systematically characterized. Future in vivo studies should validate these findings, paying particular attention to MSC retention, survival and long-term modulation of inflammatory microenvironments, with the aim of optimizing therapeutic outcomes.

## Conclusion

To summarize, the 3D microenvironment of hydrogel-embedded hG-MSCs significantly altered their functional phenotype. While metabolic activity was reduced, cell viability was largely preserved. Hydrogel embedding induced enhanced expression of IL-8, COX-2, PGE_2_, and TSG-6, while also modulating IDO-1, PD-L1, and PD-L2 expression. Furthermore, depending on the specific pro-inflammatory cytokine, the 3D microenvironment either enhanced or weakened the immune mediator response in hG-MSCs compared to conventional 2D cultures. These findings demonstrate that 3D culture systems fundamentally reshape MSC responses to inflammatory stimuli, underscoring the need to select appropriate culture models and cytokines to optimize MSC priming strategies for regenerative and immunomodulatory therapies.

## Supplementary Information

Additional file 1.

## Data Availability

All data generated or analyzed during this study are included in this published article and its supplementary information files. Additional information is available from the corresponding author upon reasonable request.

## Abbreviations

COX-2: Cyclooxygenase-2
DMEM: Dulbecco’s Modified Eagle’s Medium
DMSO: Dimethylsulfoxide
DPBS: Dulbecco's Phosphate-Buffered Saline
ELISA: Enzyme-linked immunosorbent assay
FBS: Fetal bovine serum
hG-MSCs: Human gingiva-derived mesenchymal stromal cells
hUC-MSCs: Human umbilical cord-derived mesenchymal stromal cells
IDO-1: Indoleamine-2,3-dioxygenase-1
IFN-γ: Interferon-γ
IL-1β: Interleukin-1β
IL-8: Interleukin-8
MSCs: Mesenchymal stromal cells
MTT: Thiazolyl blue tetrazolium bromide
OD450: Optical density at 470 nm
OD570: Optical density at 570 nm
PBS: Phosphate buffered saline
PD-L1: Programmed cell death ligand 1
PD-L2: Programmed cell death ligand 2
PGE2: Prostaglandin E2
PI: Propidium iodide
RT-qPCR: Reverse transcription-quantitative polymerase chain reaction
TCP: Tissue culture plastic
TDO2: Tryptophan 2,3-dioxygenase
TNF-α: Tumor necrosis factor-α
TSG-6: Tumor necrosis factor-α stimulated gene 6
YAP/TAZ: Yes-associated protein/transcriptional co-activator with PDZ-binding motif
