# Supplementary material for: 3D-embedding of human gingiva-derived mesenchymal stromal cells in “VitroGel® MSC” hydrogel alters their immune mediator expression under inflammatory conditions
Source: BMC Oral Health. 2026 May 21;26:1440. doi: 10.1186/s12903-026-08473-6 (PMC13474551; doi:10.1186/s12903-026-08473-6)
Supplement: Supplementary file 1 [file 12903_2026_8473_MOESM1_ESM.docx]

1. **Additional Files**

**Additional file 1.** Rheometric properties of “VitroGel® MSC”. “VitroGel® MSC” was mixed accordingly and loaded onto PP25/P3 measuring system. An oscillatory time sweep was performed to determine the polymerization onset (A) and gelation kinetics (B) of the hydrogel. Dynamic mechanical analysis by cyclic compression was performed to calculate the stiffness (C) and investigate the resilience (D) of the hydrogel.
